# A Tale of Two Colitides

**DOI:** 10.7759/cureus.23677

**Published:** 2022-03-31

**Authors:** Noelle Provenzano, Lindsey Forker, Lorin Berman, Paul Belser, Yogesh Govil

**Affiliations:** 1 Internal medicine, Einstein Medical Center Montgomery, East Norriton, USA; 2 Internal Medicine, Einstein Medical Center Montgomery, East Norriton, USA; 3 Pathology, Einstein Medical Center Montgomery, East Norriton, USA; 4 Gastroenterology, Einstein Medical Center Montgomery, East Norriton, USA

**Keywords:** chronic colitis, crohn’s disease (cd), inflammatory bowel disease, microscopic colitis, ulcerative colitis (uc)

## Abstract

Inflammatory bowel disease (IBD) and microscopic colitis (MC) are two distinct subgroups within the larger group of colitides. MC could manifest as collagenous colitis (CC) or lymphocytic colitis (LC). The co-occurrence of MC in patients with IBD is rare, with few cases reported. No concurrent case of MC and ulcerative colitis (UC) each presenting with distinct clinical manifestations was found in the literature review. We report a case of a 76-year-old male presenting with concurrent CC and UC. The patient's initial flare of UC was characterized by episodes of bloody diarrhea while his flare of CC was evidenced by watery diarrhea.

## Introduction

Inflammatory bowel disease (IBD) and microscopic colitis (MC) are two distinct subgroups within the larger group of colitides. IBD consists of ulcerative colitis (UC) and Crohn's disease (CD). IBD is characterized by bloody bowel movements, abdominal pain, and fever. It typically presents earlier in life, generally at the age 20-40 years. Gross examination on colonoscopy generally reveals mucosal inflammation [1]. MC generally presents later in life, typically at age 60-70 years. The general clinical presentation is characterized by watery diarrhea alone [2]. Gross examination on colonoscopy generally demonstrates a grossly normal-appearing colon. The occurrence of MC histopathologically and clinically in patients with IBD is rare, with few cases previously reported [3-6]. We report a case of a 76-year-old male patient presenting with concurrent collagenous colitis (CC) and UC. This article was previously presented as an abstract at the 2021 ACP southeastern regional posters day and doctors dilemma in Philadelphia, PA on October 23, 2021. 

## Case presentation

**Figure 1:**
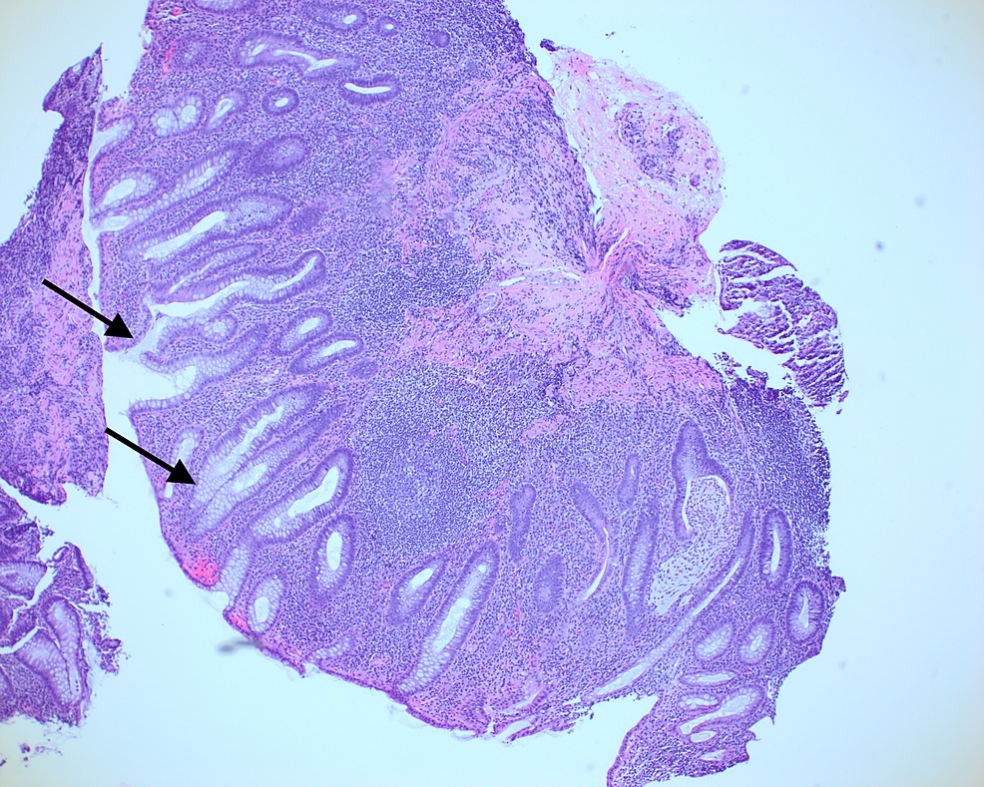
Left colon biopsy from 2019. This is a hematoxyloin and eosin stain showing glandular architectural distortion, crypt abscess formation, and increased lamina propria lymphocytes and plasma cells with basilar accentuation demonstrating inflammatory bowel disease.

The patient is a 76-year-old male with a medical history of iron deficiency anemia, hyperlipidemia, and UC who presented to the gastroenterologist with frequent loose stools. He reported six to eight non-bloody, non-formed bowel movements a day without abdominal pain. He was originally diagnosed with UC in 2019 after presenting with bloody diarrhea. His colonoscopy in 2019 showed generalized diffuse inflammation of the colon and rectal mucosa consistent with UC. Biopsies of the colon in 2019 showed changes in active chronic colitis including cryptitis, crypt abscess formation, increased lamina propria, lymphocytes, plasma cells, and background glandular architectural distortion (Figure 1).

He was treated with oral and rectal 5-aminosalicylic acid (5-ASA) with the resolution of his symptoms. His rectal 5-ASA was discontinued and he was continued on oral 5-ASA as maintenance therapy. A surveillance colonoscopy with repeat biopsies done in 2020 showed normal mucosa and no pathological changes of active UC.

**Figure 2:**
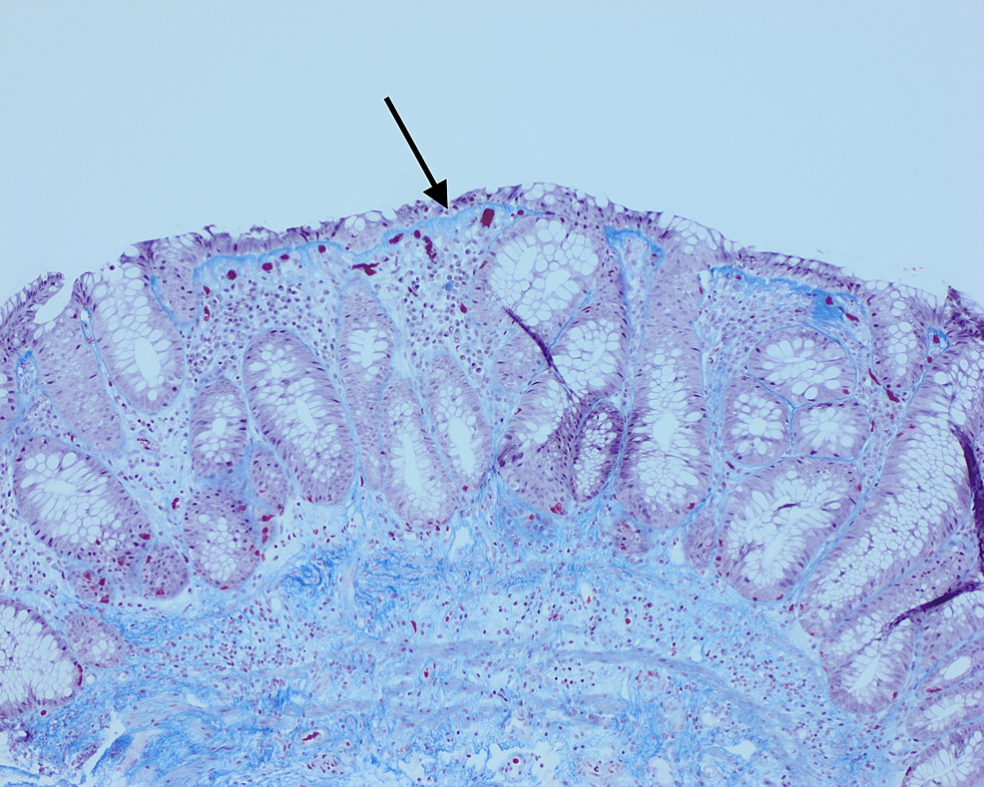
Sigmoid colon biopsy from 2021 with Masson Trichrome stain showing mildly thickened subepithelial collagen band, irregular at its base, with entrapped small capillary vessels and red blood cells. Focal surface epithelial attenuation and damage are noted as a mild background glandular architectural distortion from the quiescent inflammatory bowel disease.

Given his history, his current presentation was initially thought to be due to a flare-up of UC. To further assess a C-reactive protein, fecal procalcitonin and stool studies for *Clostridium difficile* were ordered. His C-reactive protein was <0.02 mg/L, fecal calprotectin on a stool specimen was 40 mcg/g, and *C. difficile* polymerase chain reaction (PCR) was negative. The patient was then started on oral prednisone as treatment for UC flare. He underwent a flexible sigmoidoscopy. While the sigmoid colon mucosa looked grossly normal, the biopsies revealed changed histology. The biopsies from his sigmoid colon demonstrated glandular architectural distortion, Paneth cell metaplasia, and mildly increased lamina propria lymphocytes/plasma cells consistent with quiescent chronic colitis consistent with his known history of UC. However, on the trichrome stain there was mild surface epithelial attenuation and a mildly thickened, irregular subepithelial collagen band which was suggestive of CC (Figure 2).

**Figure 3:**
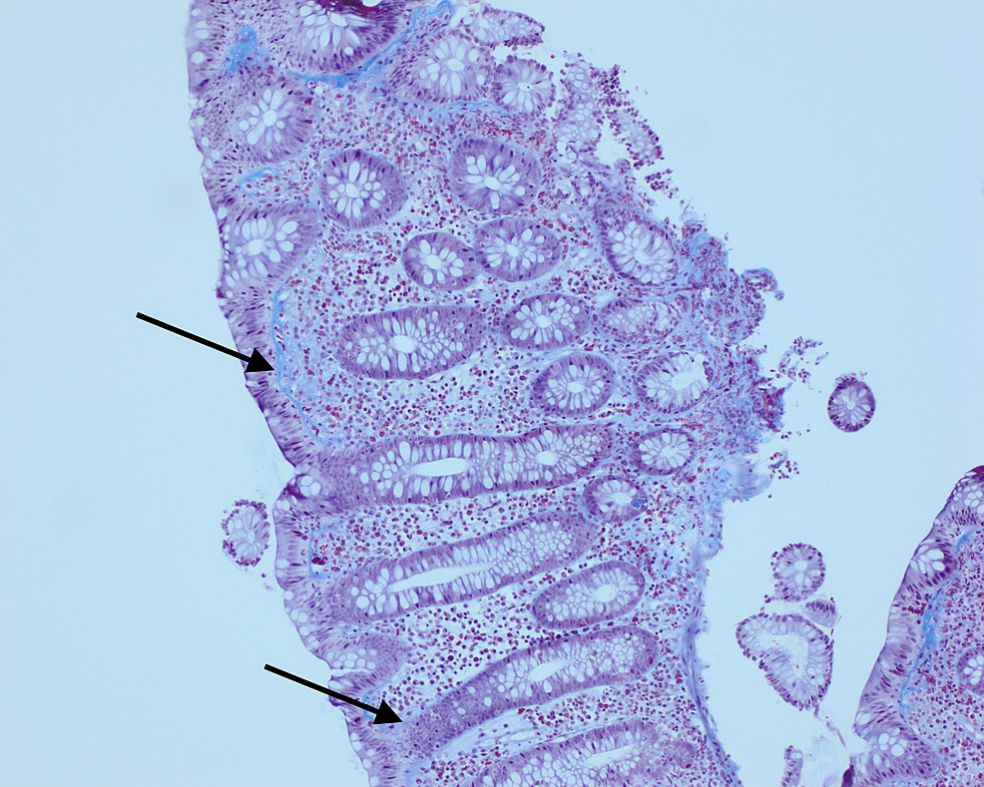
Right colon biopsy from 2019 with trichrome stain showing UC and CC. UC, ulcerative colitis; CC, collagenous colitis

His rectal biopsies again showed focal, minimal cryptitis, minimal background glandular architectural distortion, and mildly increased lamina propria lymphocytes/plasma cells. The rectal biopsies also showed patchy increased surface intraepithelial lymphocytes along with some minimal surface epithelial damage, and a mildly irregular subepithelial collagen band. The rectal biopsies were also consistent with quiescent chronic colitis accompanied by lymphocytic colitis (LC)/CC. The unique pathology of UC with collagenous colitis prompted a further review of his biopsies from 2019. With the advantage of hindsight and re-examination of his original biopsies, there was evidence of intraepithelial lymphocytes and a thickened irregular subepithelial collagenous band indicating the presence of CC with concurrent UC at that time as well (Figure 3).

After his recent biopsy results, prednisone and oral 5-ASA were discontinued and he was started on oral budesonide 9 mg daily with the resolution of his symptoms.

## Discussion

Although the onset of CC in patients with previous UC has been reported occasionally and vice versa, no case with concurrent CC and UC presenting with distinct clinical manifestations and histological manifestations was found following a thorough PubMed literature review [3-6]. The patient's initial flare-up of UC was characterized by episodes of bloody diarrhea while his flare of CC was evidenced by watery diarrhea. 

The distinction between UC and CC is important for many reasons including treatment and complications associated with each diagnosis. The initial treatment for UC is 5-ASA and corticosteroids, mainly prednisone [1]. The mainstay of treatment for CC is budesonide [7]. CC is generally benign but has been rarely associated with protein-losing enteropathy, colonic ulcerations, and spontaneous peritonitis [8]. UC on the other hand has been associated with other serious gastrointestinal comorbidities including toxic megacolon, *C. difficile* infection, chronic pancreatitis, primary sclerosing cholangitis, gallstones, autoimmune hepatitis, primary biliary cholangitis, and colon cancer. UC is also associated with extraintestinal manifestations including ankylosing spondylitis, erythema nodosum, pyoderma gangrenosum, aphthous stomatitis, and nutritional deficiencies [9]. Why and how CC develops in existing UC is not known. It has been previously hypothesized that CC development in UC patients could occur either be due to an ongoing aberrant or exaggerated healing due to an imbalance between inflammatory pathways versus mucosal healing pathways. UC is also associated with extraintestinal manifestations including ankylosing spondylitis, erythema nodosum, pyoderma gangrenosum, aphthous stomatitis, and nutritional deficiencies [9]. Once UC is controlled with treatment the CC could manifest.

## Conclusions

This patient presented with two distinct histological and clinical manifestations of MC and UC, an occurrence that has not been reported in detail. Although, these are likely random associations of two different disorders, our case highlights that MC should be considered in the patient with new-onset watery diarrhea or non-formed stools after successful treatment of UC. This should especially be considered in cases with complete mucosal healing on surveillance colonoscopies. It is important to consider appropriate medication adjustment and repeat biopsies with this clinical change. 
